# Design and analysis of stably integrated reporters for inducible transgene expression in human T cells and CAR NK-cell lines

**DOI:** 10.1186/s12920-019-0489-4

**Published:** 2019-03-13

**Authors:** Sergey V. Kulemzin, Daria A. Matvienko, Artur H. Sabirov, Arpine M. Sokratyan, Daria S. Chernikova, Tatyana N. Belovezhets, Anton N. Chikaev, Aleksandr V. Taranin, Andrey A. Gorchakov

**Affiliations:** 1 Institute of Molecular and Cellular Biology SB RAS, Novosibirsk, Russia; 2 Novosibirsk State University, Novosibirsk, Russia

**Keywords:** Chimeric antigen receptor, Cancer, Reporter, Inducible promoter, NK cell lines

## Abstract

**Background:**

Cytotoxic activity of T- and NK-cells can be efficiently retargeted against cancer cells using chimeric antigen receptors (CARs) and rTCRs. In the context of solid cancers, use of armored CAR T- and NK cells secreting additional anti-cancer molecules such as cytokines, chemokines, antibodies, BiTEs, inverted cytokine receptors, and checkpoint inhibitors, appears particularly promising, as this may help overcome immunosuppressive tumor microenvironment, attract bystander immune cells, and boost CAR T/NK-cell persistence. Placing the expression of such molecules under the transcriptional control downstream of CAR-mediated T/NK-cell activation offers the advantage of targeted delivery, high local concentration, and reduced toxicity. Several canonic DNA sequences that are known to function as activation-inducible promoters in human T and B cells have been described to date and typically encompass the multimers of NFkB and NFAT binding sites. However, relatively little is known about the DNA sequences that may function as activation-driven switches in the context of NK cells. We set out to compare the functionality of several activation-inducible promoters in primary human T cells, as well as in NK cell lines NK-92 and YT.

**Methods:**

Lentiviral constructs were engineered to express two fluorescent reporters: mCherry under 4xNFAT, 2xNFkB, 5xNFkB, 10xNFkB, 30xNFkB promoters, as well as two variants of the CD69 promoter, and copGFP under the strong constitutive promoter of the human EF1a gene. Pseudotyped lentiviral particles obtained using these constructs were transduced into primary human T cells and NK-92 and YT cell lines expressing a CAR specific for PSMA. The transgenic cells obtained were activated by CD3/CD28 beads (T cells) or via a CAR (CAR-NK cell lines). Promoter activity before and after activation was assayed using FACS analysis.

**Results:**

In T cells, the CD69 promoter encompassing CNS1 and CNS2 regions displayed the highest signal/noise ratio. Intriguingly, in the context of CAR-YT cell line neither of the seven promoters tested displayed acceptable activation profile. In CAR-NK-92 cells, the largest fold activation (which was modest) was achieved with the 10xNFkB and 30xNFkB promoters, however its expression was clearly leaky in “resting” non-activated cells.

**Conclusions:**

Unlike in T cells, the robust activation-driven inducible expression of genetic cassettes in NK cells requires unbiased genome-wide identification of promoter sequences.

**Electronic supplementary material:**

The online version of this article (10.1186/s12920-019-0489-4) contains supplementary material, which is available to authorized users.

## Background

Chimeric antigen receptors (CARs) are engineered molecules that endow the immune effector cells, typically T cells, with the ability to recognize a pre-defined surface epitope and destroy the cognate cell in an MHC-unrestricted manner. CARs are composed of the extracellular antigen-recognition module and the intracellular signaling domain joined together by the hinge and transmembrane moieties. Grafting the specificity provided by a CAR to T or NK cells allows their retargeting against cancer cells and has revolutionized the field of adoptive cellular immunotherapy, as it has allowed efficient treatment of a number of hematological malignancies. Yet, the success of CARs has not been clinically translated to solid cancers [1], and the use of so-called “armored” CAR T cells co-expressing a CAR and a cytokine or other therapeutic molecule of interest may help solve this issue by overcoming the immunosuppressive tumor microenvironment and by providing improved activity and persistence of such engineered cells [2–4]. Ideally, expression of such accessory molecules should be coupled to the CAR-mediated activation of CAR T- or NK cells, as this may help achieve local delivery, favorable pharmacokinetics profile and translate into reduced systemic toxicity [5, 6]. Also, placing a convenient reporter under the control of activation-inducible promoter is a powerful approach to develop so-called sensor cell lines that may be used for screening purposes and signaling pathway analyses.

Immune cell activation is a highly regulated process, with multiple signaling pathways converging in the induction of gene expression [7] as well as in gene repression [8]. Furthermore, additional layers of regulation of cell activation are known to occur at the level of alternative splicing [9, 10], individual mRNA turnover rate control [11–13], and at the post-translational level [14]. Although activation-associated signaling pathways in T-, B- and NK-cells are overall homologous and center around the activity of NFkB, NFAT, AP-1, and ERK transcription factors [15–18], the resulting differential expression programmes are clearly different and likely depend on the multitude of factors ranging from the pre-existing cell type-specific chromatin organization to the differences in subunit composition of the transcription factors involved. Intriguingly, many aspects of NK cell activation still remain poorly explored, given the extreme phenotypic heterogeneity of this cell type [19, 20].

In the context of T cells, CARs typically engage essentially the same molecular machinery as operates downstream of the TCR and co-activation pathways [21]. Yet, the exact events associated with CAR activation in NK cells are poorly defined, despite demonstrable functionality of CAR-NK cells [22–26]. In this study, we asked whether similarly to armored CAR T cells, armored CAR-NK cells can be created by using one of the conventional NFAT- or NFkB-based promoters. To address this question, we designed several reporter constructs that were tested in CAR-NK cell lines and T cells.

## Results

### Construct design

**Fig. 1:**
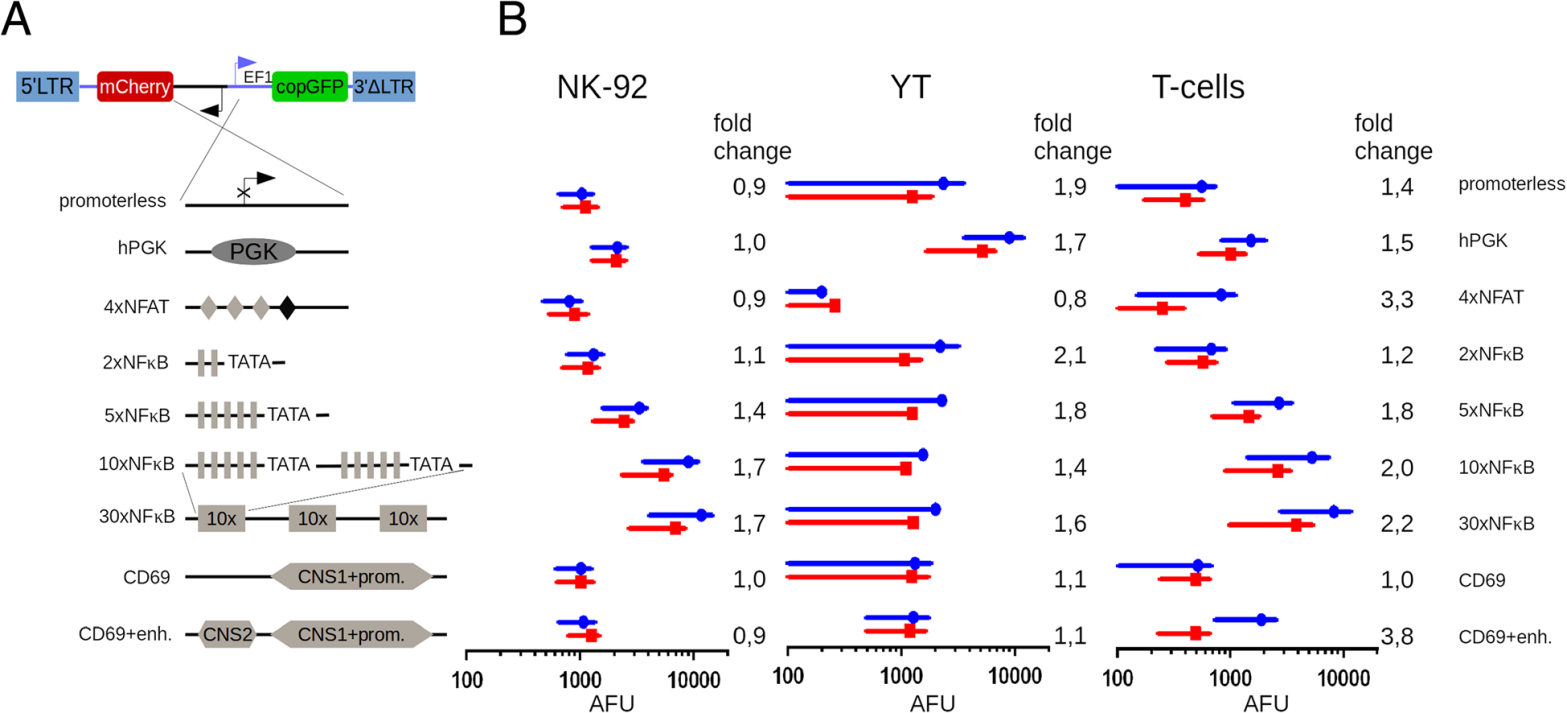
Analysis of the chromosomally integrated activation-induced promoters in CAR NK-92, CAR YT and T cells. **a** Scheme of the lentiviral vector (from 5’LTR to 3’LTR) encoding divergently transcribed copGFP and mCherry reporters driven by the constitutive EF1 promoter and one of the activation-inducible promoters, respectively. Organization of the activation-inducible promoters integrated into the genome as reporter constructs is shown below. **b** CopGFP-normalized mean fluorescence values of mCherry reporter in CAR NK-92 and CAR YT cells co-incubated with target HEK293T(PSMA) cells (blue) or isogenic controls (HEK293T, red). Similarly, the values observed for bead-activated (blue) and resting (red) T cells are shown. Mean fluorescence values are shown as circles and squares, with quartile ranges indicated by lines. Fold change of normalized mCherry signal is indicated next to each plot. AFU – arbitrary fluorescence units

To enable comparisons of activity of select promoters in the chromatin context, we chose to generate the cells and cell lines wherein the reporters would be stably integrated rather than episomal. This, in our opinion, should closer mimic the dynamics and regulation of expression once the proteins of interest are to be expressed instead of the fluorescent reporter proteins. We modified a third-generation SIN lentiviral vector, pCDH, by replacing the CMV promoter with a “promoter-mCherry” cassette inserted in an inverted orientation, so that no residual transcription from the vector 5’LTR [27, 28] would lead to the leakage of reporter in non-inducing conditions. Specifically, several synthetic promoters and promoter/enhancer combinations whose activity was expected to be inducible in the context of T and/or NK cells were tested, namely, sequences composed of the multimers of NFAT-binding sites, as well as of NFkB-binding sites. As controls, we used two constructs lacking any promoter sequences or harboring a moderately strong constitutive human PGK promoter (Fig. 1a). Importantly, all the constructs were marked with an EF1-driven copGFP expression cassette of the pCDH vector, which allowed tracking transduced cells and normalizing the mCherry reporter activity.

### NFAT- and NFkB-based reporters behave differently in primary human T cells and CAR-NK cell lines

In order to compare promoter activities in a native chromosomal context, primary human T cells, as well as human NK cell lines NK-92 and YT were transduced with the above lentiviral constructs. The NK cell lines used were pre-engineered to express a second-generation PSMA-specific CAR [29], so that their activation could be matched in the same context of CAR-dependent NK cell activation regardless of the differences in expression of activating and inhibitory receptors as well as signaling details. Expression of the copGFP reporter in transduced cells was used for cell gating in downstream FACS analyses.

T cells were activated using CD3-CD28-coated beads, as this represents an easily tractable and physiological way for T cell activation. In turn, to activate CAR-YT and CAR-NK-92 cells, they were co-incubated with target HEK293T-PSMA cells. Fluorescence levels of the mCherry reporter normalized by the copGFP fluorescence were then measured in resting and activated cells to quantify relative activity and fold induction of the promoters tested. Both in the primary T cells and in CAR-NK-92 cells, the strongest mCherry expression following activation was detectable for the 30xNFkB promoter. Notably, this promoter was also the leakiest and displayed the highest background mCherry production in the absence of activation, with about 2-fold induction level (Fig. 1b). 10xNFkB promoter was overall similar, and showed somewhat reduced expression levels. 5xNFkB element was significantly weaker, with 2xNFkB cassette showing little if any promoter activity. Surprisingly, reporter expression from the 4xNFAT promoter was very modest, yet it provided decent fold induction in primary T cells, unlike in CAR-NK-92 cells where it displayed negligible activity. Despite pronounced cell activation, as inferred from IFN-g secretion (data not shown) and surface expression of the degranulation marker CD107a [30] (Additional File 1: Figure S1), neither of the promoters tested were active in the context of CAR-YT cells.

**Fig. 2:**
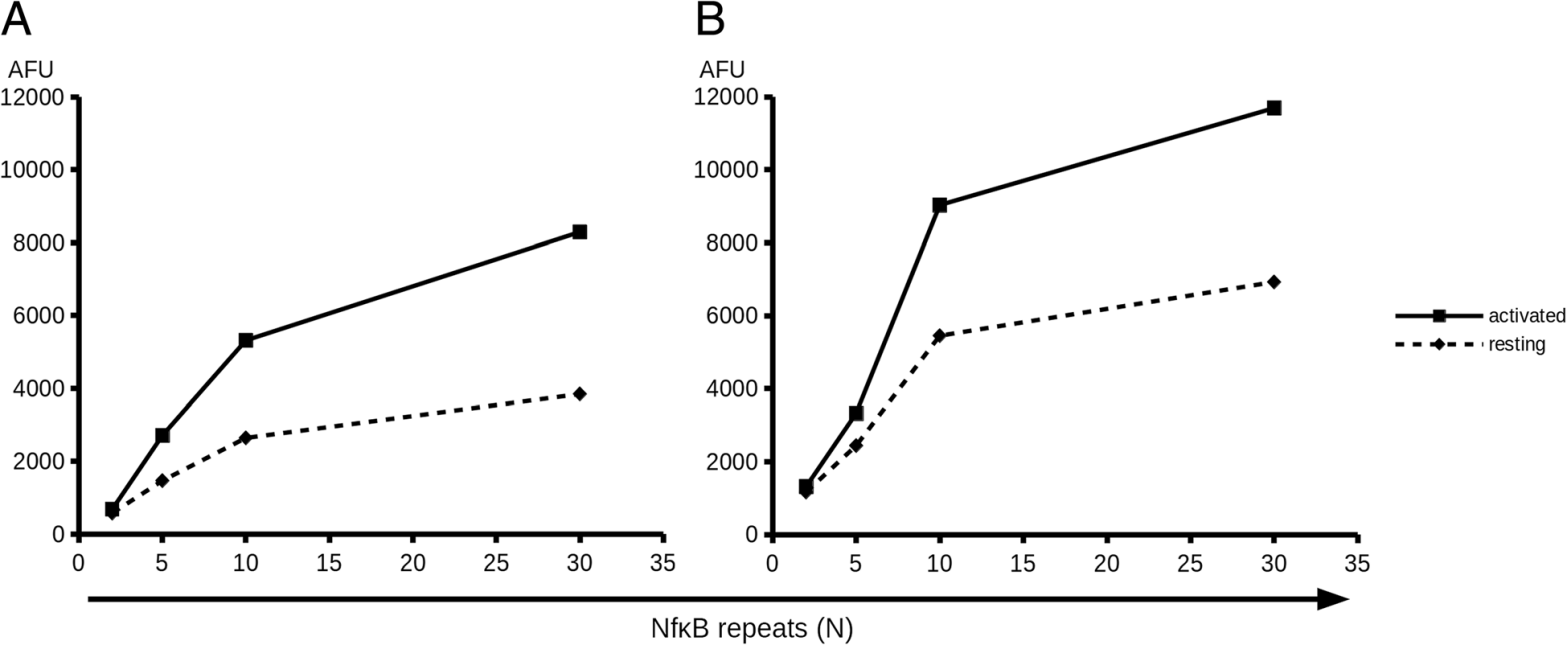
Dependence of inducible reporter expression on the number of NFkB binding sites present in the promoter cassette in primary human T cells (**a**) and CAR NK-92 cells (**b**). Relative mCherry fluorescence values (copGFP-normalized) are shown as solid lines (15 h post activation) and dashed lines (T cells incubated for 15 h without beads or CAR NK-92 cells incubated with PSMA-negative non-target HEK293T cells). AFU – arbitrary fluorescence units

To understand how many NFkB multimers are needed to provide optimal activation-induced reporter expression, we varied the number of NFkB binding sites in our constructs from 2 to 30 and analyzed the promoter activity at baseline and upon activation (Fig. 2). For T and CAR NK-92 cells, fluorescent reporter signal grows nearly linearly as the number of NFkB binding sites increases from 2 to 10. Further addition of NFkB binding sites has little influence on the reporter activity. Thus, for these cells, promoter element composed of a multimer of 10 NFkB binding sites appears optimal as it provides the widest dynamic range.

### Regulatory regions of the human CD69 gene as an activation-inducible promoter for T- and CAR NK-cells

**Fig. 3:**
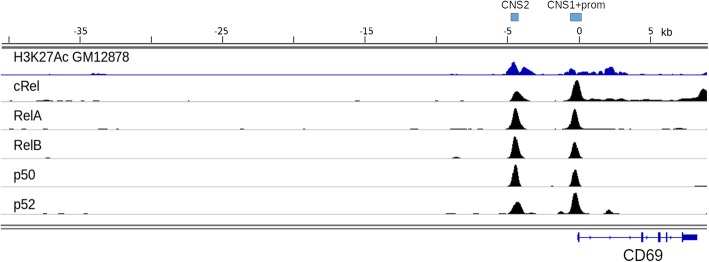
Analysis of regulatory elements present within 40 kb upstream of the human CD69 gene. Genomic coordinates are set to zero at the start codon of CD69. Profiles for the H3K27ac histone mark (dark blue) and NFkB family members (black) available for the B cell line GM12878 are shown. CD69 gene exon-intron structure is provided below. Light blue bars depicted above the genomic scale correspond to the regions used for constructing activation-inducible constructs CD69prom + enh and CD69prom

CD69 is known as one of the early markers of B-, T-, and NK-cell activation [31–35]. We asked whether promoter/enhancer sequences of CD69 could function as activation-inducible promoters in T- and NK-cells. Four highly conserved non-coding regions referred to as CNS1–4 located within 50 kb upstream of the mouse CD69 promoter were previously identified as contributing to the developmental and temporal control of CD69 activation in T- and B- cells [36]. Intriguingly, later study from the same group indicated that CNS2 region found some 5 kb upstream of the mCD69 TSS behaved as a potent enhancer, at least in the context of episomal reporter assays [37]. Using the publicly available ChIP-seq data for H3K27ac (active enhancer mark) [38] and NFkB family members (RelA, RelB, cRel, p52, p50) [39] in constitutively active EBV-transformed human lymphoblastoid cell line GM12878, we focused our analysis on the region spanning 5 kb upstream of the human CD69 gene and observed that the binding profiles for NFkB subunits as well as H3K27ac are nearly identical, with enrichment peaks mapping to the promoter region, first intron, and CNS2 enhancer of hCD69 (Fig. 3). Two reporters encompassing CNS1 + promoter or CNS2 + CNS1 + promoter elements were created and introduced into primary human T cells and CAR-NK cell lines via lentiviral transduction. Predictably, minimal hCD69 promoter combined with the CNS1 element showed no promoter activity in either primary human T cells or CAR-NK cell lines (Fig. 1b). In contrast, addition of the CNS2 element converted this construct into an activation-inducible reporter with a higher “signal/noise” expression ratio, although this effect was restricted to primary human T cells.

**Fig. 4:**
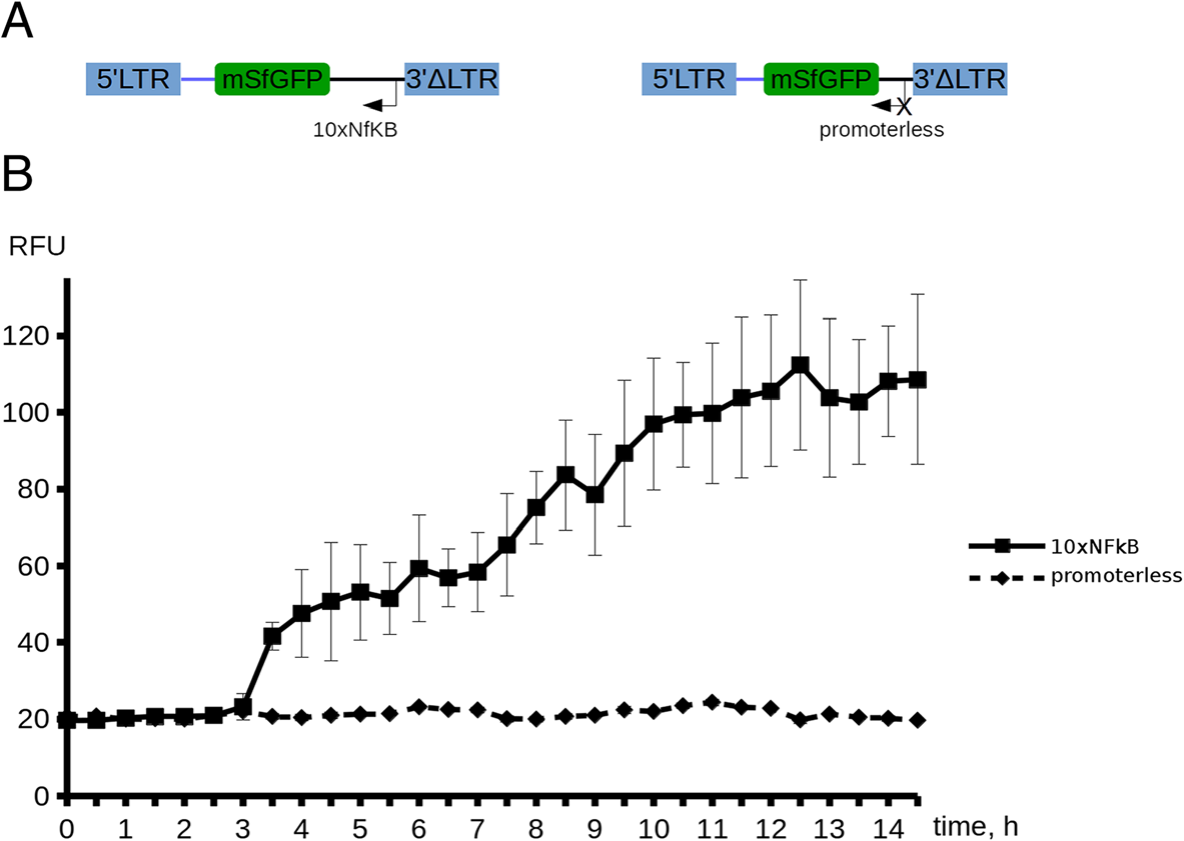
Real-time fluorescence microscopy analysis of activity of the activation-inducible promoter 10xNFkB in Jurkat T cells. (**а**) Structure of the lentiviral constructs used for transduction. 10xNFkB-sfGFP module is oriented antisense relative to the 5’LTR-3’LTR of the vector. (**b**) Mean cell fluorescence values are plotted for the 10xNFkB-bearing and promoterless (neg. control) construct in the CD3/CD28-activated transduced cells as a function of time. Dynamics of the fluorescence signal in unstimulated 10xNFkB Jurkat cells is not shown on the plot, as it was indistinguishable from the control

For translational applications, identification of activation-inducible promoters that respond to cell activation the soonest is of particular interest, as this may afford production of the protein of interest shortly after induction. Of the promoters tested in our study, 10xNFkB and 30хNFkB cassettes potently drive reporter expression at comparable levels, so we used the shorter 10xNFkB promoter for time-course microscopy analysis of reporter activation in vitro. We chose to redesign this reporter by removing EF1-copGFP cassette and fusing the 10xNFkB element with a cell membrane-anchored fast-maturing sfGFP (Fig. 4a). Jurkat cells transduced to express 10xNFkB-sfGFP were activated with CD3/CD28 beads, and immediately subjected to time-lapse fluorescence imaging for 15 h to monitor fluorescence dynamics. At ~ 3.5 h timepoint, significant increase in sfGFP signal was readily detectable, which continued to grow linearly until reaching a plateau at 10 h post induction (Fig. 4b). Maximum ratio of the fluorescence signal values acquired in activated/baseline conditions reached the value of ~ 5 (at 10 h timepoint). Jurkat cells transduced with a construct lacking a promoter cassette served as a negative control.

## Discussion

Functionality of inducible promoters is primarily assessed by measuring two parameters, namely absolute expression levels and expression ratios post- and pre-induction, which is analogous to signal-noise ratio. Selecting a potent promoter is indispensable for high-level expression of the proteins of interest, such as therapeutic antibodies and growth factors. Yet, high expression may not be as important as the lack of leaky target protein expression at baseline. Particularly, this may be the case whenever toxic or highly biologically active molecules need to be expressed, whose baseline expression must be tightly shut.

In the present study, we report on the design and analysis of two types of activation-inducible promoters functional in primary human T cells. One type, exemplified by the 10хNFkB and 30хNFkB elements, shows robust expression in the activated T cells at the level about an order of magnitude higher than what is provided by the constitutive hPGK promoter. This is however accompanied with a relatively high background expression. The other type, represented by the moderately strong CNS2 + CNS1 + promoter, shows very little to no reporter expression in non-activated T cells. Of these promoters, the broad dynamic range of activation provided by the 10хNFkB element suggests that it can be used for TCR- or CAR-dependent gene expression in the context of primary human T cells.

No promoters tested in this study displayed the desirable combination of high fold induction and low baseline expression in NK cell lines YT and NK-92. Notably, for NK-92 cells, 10хNFkB and 30хNFkB mediated high CAR-dependent reporter expression, which was accompanied with high level of promoter activity in non-induced conditions, similar to T cells.

Our observation that NFAT-based promoter was inefficient in the context of NK cell lines appears somewhat surprising given the well-established positive role of NFAT in T- and B-cell activation, yet this was not unexpected. In fact, it has recently been demonstrated that NFAT-dependent signaling is dispensable for NK cell activation and is furthermore associated with negative regulation of NK cell function [40]. It is presently unclear why NFkB-based promoters are dysfunctional in CAR-YT cells. Compared, to NK-92 cells, YT cell line is poorly characterized in terms of signaling details and expression. Previously, we and others reported that these cells can undergo activation driven by the first-generation [41] and second-generation CARs [24] and kill appropriate target cells. Whether this activity is dependent on NFkB-controlled gene expression and which pathways are involved is presently unknown, warranting further investigation.

Leaky expression of NFkB-based promoters in NK-92 cell background is likely attributable to the fact that this IL-2 dependent cell line is partially activated even in the absence of stimuli from other cytokines or cell surface targets [42]. Similarly, NFkB promoters are also expected to be leaky in primary human T cells because efficient lentiviral delivery into T cells requires their prior activation and further supplementation with IL-2 to expand the transduced cell population, i.e. some residual level of activation of T cells may account for the observed effect.

Native gene promoters and enhancers typically encompass clusters of multiple transcription factor binding sites, providing expression robustness and fine-tuned control [43, 44]. This is in contrast to the monomorphic nature of artificial activation-inducible promoters used in our work. Hence, we turned to the CD69 promoter to test the functionality of an endogenous promoter in our system. Our analysis indicated that in the context of T cells this promoter appears on par with the NFAT-based promoter, and that it is not prone to leaky expression, as are the NFkB-based promoters. It was previously reported that synthetic NFAT promoter could be successfully applied for inducible transgene expression in the context of CAR T-cells [5, 6]; it remains to be explored whether more clinically relevant secretion dynamics can be achieved with CD69-based promoter(s).

Numerous binding sites of transcription factors from NFAT, NFkB, EP300, and other families are present in the promoter and CNS2 enhancer region of CD69, as inferred from the published ChIP-seq datasets [45] and earlier analysis [37]. Which of the signaling cascades has a leading role in driving the expression from this promoter is not known. In all likelihood, the contribution of NFkB pathway is minimal here, as it functions well in CAR NK-92 cells, whereas the CD69 promoter variants tested in these cells display no activity.

Finally, we note that the construct expressing membrane-bound sfGFP under the 10xNFkB promoter appears promising for designing improved CAR variants. This approach appears particularly attractive in the context of CAR display, as one can co-express 10xNFkB-sfGFP reporter with a library of CARs encompassing various scFvs [46], hinge or signaling domains [47], and measure the activation of such reporter cells immediately after their contact with target cells in a native format. We furthermore envisage that this or similar reporter construct(s) should greatly simplify the screening protocol and make it more cost-effective, as it obviates the need for staining the cells with conjugates against activation markers and allows selection of the cells according to the magnitude of activation provided by the CAR.

## Conclusion

Our data indicate that in the context of primary T cells, activation-inducible CD69 promoter variant provides the highest fold induction. This promoter therefore can be used for expressing proteins in the activated, but not resting human T or CAR T cells. The most potent inducible promoter in our assays, 10xNFkB, performs equally well in T and NK cells and features significant baseline activity in the absence of activation. When driven by this promoter, expression of the protein of interest is detectable as early as 4 h following cell activation. Unfortunately, our efforts failed to uncover the activation-inducible promoter configuration that would provide efficient expression in activated NK cells combined with low background activity in non-stimulated NK cells. Thus, unbiased approaches based on the genome-wide analysis of promoter elements are needed to identify regulatory elements that would drive efficient CAR-dependent expression in NK cells.

## Methods

### Plasmid construction

Lentiviral pCDH vector (CD-511, SysBio) was used as a backbone to clone the series of reporter constructs. First, ClaI-XbaI fragment encompassing the CMV promoter was removed from pCDH and replaced with a ClaI/XbaI-flanked TatRRR-mCherry-NLS fragment obtained by PCR on mPB-L3-ERT2.TatRRR-mCherry plasmid [48] using the primers mCherryXbaF ggttctagagccgccaccatgtatggcaggaagaagcg and mCherryClaR atggaatcgatttattcaaagattacttgtacagctcgtccatg. In the pCDH* vector thus obtained (control “promoterless” construct), mCherry reporter is found in an antisense orientation relatively to the constitutive EF1 promoter from which it is separated by a multiple cloning site. BamHI/XbaI- flanked PCR products containing 4xNFAT-IL2min promoter (hereafter, 4xNFAT) from the 4xNFAT-d2EGFP plasmid [49] or 5xNFkB-minHIVtata promoter (hereafter, 5xNFkB) from the Lenti-NF-Gluc plasmid [50] were amplified using the primer pairs: NFATBamF 5′-tttggatccgttttctgagttacttttgtatc-3′, NFATXbaR 5′-cactctagagggcaggagttgaggttact-3′, and NFkBBamF 5′-tctggatccgtttgaagatcttggg-3′, NFkBXbaR 5′-ccatctagacaccacactggactagtggatt-3′, respectively, and ligated into pCDH* digested with BamHI and XbaI. In the case of 5xNFkB-based promoter, clones containing 2, 10, 20 and 30 repeats of NFkB binding site were obtained as a by-product of ligation. Human PGK promoter was inserted between AgeI and XbaI sites of pCDH* as a 512-bp PCR product (hPGKAgeF 5′-tctaccggtcggggttggggttgcg-3′, hPGKXbaR 5′-ggatctagatggggagagaggtcggtgat-3′). Finally, two constructs containing CNS1 or CNS1 + CNS2 upstream regulatory elements of the human CD69 promoter were obtained by sequential cloning of the BamHI/NheI-flanked CNS1 region (1574 bp, Cd69BamF 5′-gtgggatccgaagagtgagtcggttaaag-3′, Cd69NheR 5′-atcgctagctcaagattccctagttaat-3′) and BamHI/NotI-flanked CNS2 region (1040 bp, CD69NotF 5′-tacaattgcggccgctttatgatagcatagtagccca-3′, CD69BamR 5′-ttggatccagggagacattttatgtgtc-3′). Human genomic DNA was used as a template for PCR to generate PGK and CD69 promoter cassettes.

### Cell culture

HEK293T, Jurkat, and NK-92 cell lines were purchased from ATCC. YT cell line was a kind gift of Dr. A.V. Filatov. Primary human T cells were isolated from peripheral blood of a healthy donor who provided written informed consent in accordance with the approval of the Ethics Committee on Animal and Human Research of the Institute. T cells and cell lines were grown in IMDM (ThermoFisher) supplemented with 10% FCS (15% for NK-92), 100 μg/ml penicillin and 100 μg/ml streptomycin, at 37 °С in a humidified atmosphere of 5% CO2. IL-2 (200 μg/ml) was included into growth media for NK-92 cell cultivation.

### Lentiviral assembly and transduction

Lentiviral particles were produced as described previously [24]. Briefly, HEK293T cells were transfected with a mixture of pMD2.G, psPAX2 and gene transfer plasmid using calcium-phosphate transfection protocol. Two days later, the supernatants of conditioned media were filtered and used for cell transduction either immediately or following ultracetrifugation. Cells were transduced using spinoculation protocol in the presence of 8 μg/ml polybrene (YT, NK-92, and Jurkat cells) or 10 μg/ml protamine sulphate (pre-activated primary human Т cells). Cells were subjected to functional tests no sooner than 10 days after transduction. CAR-NK cell lines were obtained from NK-92 and YT cells by transducing a second-generation PSMA-specific CAR of the following structure: SP (mIgK)-scFv(J591)-IgG1hinge (CH2-CH3) - CD28TM-CD28cyto-CD3z(cyto) [29].

### Activation analysis

Functional analysis of the promoter cassettes was performed by activating transduced primary T cells and CAR-NK cells for 16 h. T cells were activated with Dynabeads Human T-Activator CD3/CD28 (Thermo Fisher), at a 1:1 cell:beads ratio. Cells and beads were co-incubated in 24-well plates at a density of 1 million cells per well. Control transduced T cells were incubated in parallel at the same density without adding beads. CAR-NK cells were incubated 1:1 with target HEK293T-PSMA cells. As an isogenic negative control, CAR-NK cells were incubated with HEK293Т cells. To account for varying transduction efficiency, only copGFP-positive cells were taken into analysis. Reporter fluorescence was measured using Sony SH800 flow cytometer. Mean mCherry fluorescence (driven by the activation-inducible promoter) was normalized to the copGFP signal (driven by the constitutive EF1a promoter), in order to factor in the differences in fluorescence associated with altered cell size and general increase in transcription background following cell activation.

### Real-time cell imaging

To monitor the activation properties of the 10xNFkB element in transduced Jurkat cells, they were mixed with Dynabeads Human T-Activator CD3/CD28 (ThermoFisher) at a 1:1 ratio and immediately placed into the Cell-IQ imaging and analysis system (Chip-Man Technologies). Cells were imaged under a phase contrast and fluorescence microscopy regimens for 16 h. Next, average fluorescence values of the cells on each image was calculated using Icy platform [51]. Data on the average fluorescence of the cell population at any given moment of time was inferred from the imaging data for 20–50 cells per field.

## Additional file

Additional file 1: **Figure S1.** CAR NK-92 (**A**) and CAR-YT (**B**) cells become activated upon 4 h incubation with target HEK293T-PSMA cells and up-regulate the degranulation marker CD107a on the surface. FACS plots for the resting CAR-NK cells (light gray) and activated CAR-NK cells (dark grey) are shown. (**С**) Activation of primary human T cells 4 h following addition with CD3/CD28 beads. Cells were immunostained with anti-CD69 conjugates and analyzed by FACS. Resting T cells (light gray), activated T cells (dark grey). (PNG 89 kb)

## Abbreviations

BiTE: Bispecific T cell engager
CAR: Chimeric antigen receptor
ChIP-seq: Chromatin immunoprecipitation sequencing
CNS: Conserved non-coding sequence
FACS: Fluorescence activated cell sorting
NK: Natural killer
TCR: T cell receptor
TSS: Transcription start site
