# Supplementary figures and images for: Design and analysis of stably integrated reporters for inducible transgene expression in human T cells and CAR NK-cell lines
Source: BMC Med Genomics. 2019 Mar 13;12(Suppl 2):44. doi: 10.1186/s12920-019-0489-4 (PMC6417161; doi:10.1186/s12920-019-0489-4)

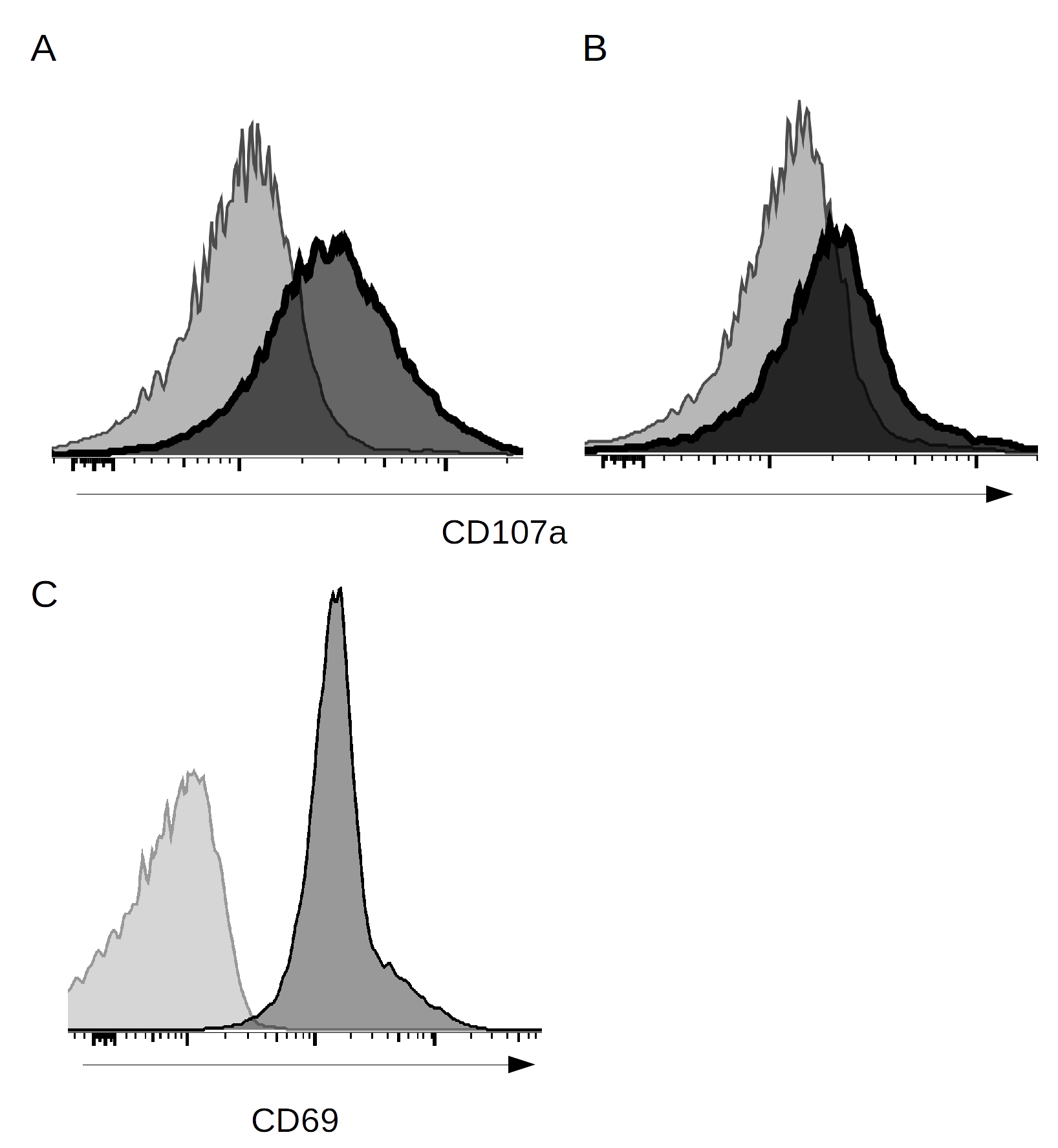

Supplement: Supplementary file 1 — Figure S1. CAR NK-92 (A) and CAR-YT (B) cells become activated upon 4 h incubation with target HEK293T-PSMA cells and up-regulate the degranulation marker CD107a on the surface. FACS plots for the resting CAR-NK cells (light gray) and activated CAR-NK cells (dark grey) are shown. (С) Activation of primary human T cells 4 h following addition with CD3/CD28 beads. Cells were immunostained with anti-CD69 conjugates and analyzed by FACS. Resting T cells (light gray), activated T cells (dark grey). (PNG 89 kb) [file 12920_2019_489_MOESM1_ESM.png]
